# A Single Amino Acid Substitution in Structural Protein VP2 Abrogates the Neurotropism of Enterovirus A-71 in Mice

**DOI:** 10.3389/fmicb.2022.821976

**Published:** 2022-03-17

**Authors:** Huimin Yeo, Connie Wan Hui Chong, Elijah Weihua Chen, Ze Qin Lim, Qing Yong Ng, Benedict Yan, Justin Jang Hann Chu, Vincent T. K. Chow, Sylvie Alonso

**Affiliations:** ^1^Infectious Diseases Translational Research Programme, Department of Microbiology and Immunology, Yong Loo Lin School of Medicine, National University of Singapore, Singapore, Singapore; ^2^Immunology Programme, Life Sciences Institute, National University of Singapore, Singapore, Singapore; ^3^Department of Laboratory Medicine, Molecular Diagnosis Centre, National University Health System, Singapore, Singapore; ^4^Institute of Molecular and Cell Biology, Agency for Science, Technology and Research (A*STAR), Singapore, Singapore

**Keywords:** foot and mouth disease (HFMD), enterovirus A-71, SCARB2, VP2 EF loop, neurovirulence

## Abstract

Enterovirus 71 (EV-A71) causes hand, foot, and mouth disease (HFMD) in children and has been associated with neurological complications. With no specific treatment and a monovalent vaccine limited to the Chinese market, HFMD remains a serious public health concern and an economic burden to affected societies. The molecular mechanisms underpinning EV-A71 neurovirulence have yet to be fully elucidated. In this work, we provide experimental evidence that a single amino acid substitution (I to K) at position 149 in structural protein VP2 of a non-mouse-adapted EV-A71 strain completely and specifically abrogated its infectivity in murine motor neuron-like NSC-34 cells. We showed that VP2 I149K mutant was impaired in murine SCARB2-mediated entry step but retained the ability to attach at the cell surface. *In vivo*, VP2 I149K mutant was fully attenuated in a symptomatic mouse model of progressive limb paralysis. While viral titers in limb muscles were comparable to mice infected with parental wild-type strain, significantly lower viral titers were measured in the spinal cord and brain, with minimal tissue damage, therefore indicating that VP2 I149K mutant is specifically impaired in its ability to invade the central nervous system (CNS). This study highlights the key role of amino acid at position 149 in VP2 in EV-A71 neurovirulence, and lends further support that the EF loop of VP2 represents a potential therapeutic target.

## Introduction

Hand, foot, and mouth disease (HFMD) is a common viral infection that affects mostly infants and children below 5 years of age.^1^ The main causative agents responsible for HFMD belong to the *Picornaviridae* family, and consist predominantly of coxsackievirus type A (CA) strains (CA16 and CA6), and enterovirus 71 (EV-A71) (Mao et al., 2014; Bian et al., 2015; Nassef et al., 2015). HFMD occurs worldwide but is more prevalent in the Western Pacific region (China, Taiwan, Malaysia, Singapore, Japan, and Australia) where recurrent cyclical outbreaks and large-scale epidemics have been reported (Koh et al., 2016).

HFMD is highly contagious and is usually transmitted via the oral-fecal route. Clinical manifestations are generally mild and self-limiting, and include fever, sore throat, and vesicular eruptions on the hands, feet, and oral mucosa (Nassef et al., 2015). However, more severe clinical manifestations with neurological complications have been associated with EV-A71 infection and can be fatal (Huang et al., 1999). These manifestations include aseptic meningitis, brainstem encephalitis, acute flaccid paralysis, and cardiopulmonary dysfunction. When not fatal, the involvement of the central nervous system (CNS) during EV-A71 infection may lead to long-term cognitive and motor disorders (Huang et al., 2006; Chang et al., 2007). The average time to full recovery from uncomplicated HFMD is 7–10 days, but an infected individual can remain contagious to other people (e.g., siblings, classmates.) for up to 6 weeks, long after apparent recovery from symptoms (Teng et al., 2013). This further contributes to sustained transmission among susceptible individuals.

With no therapeutic intervention available so far, treatment is primarily supportive. Epidemic control measures have therefore been instrumental in preventing virus transmission among individuals and limiting contact with contaminated surfaces (Chan et al., 2017). Three EV-A71 formalin-inactivated whole virus vaccine formulations have been approved by the Chinese Food and Drug Administration (CFDA) and have been launched in China (Mao et al., 2016; Yi et al., 2017). These vaccines are, however, far from ideal, as they include a single EV-A71 genotype (C4) that may confer limited or transient cross-protection against the other genotypes. Furthermore, a multiple-dose regimen is required to sustain the protective antibody responses.

The disease outcome in EV-A71 patients results from the interplay between the patient’s immune status and genetic make-up, as well as the intrinsic virulence of the infecting EV-A71 strain. Studies have compared genome sequences from various EV-A71 strains isolated from patients displaying various disease severities, in an attempt to establish correlations between genome sequence and disease severity (Wang et al., 2012; Jia et al., 2016). Numerous studies have reported the role of specific amino acids or non-coding nucleotide sequences in receptor-mediated cell tropism, fitness and/or *in vivo* virulence of EV-A71, which have improved our understanding of the molecular determinants involved in EV-A71 pathogenesis, with important implications for therapeutic and vaccine design strategies (Wang and Li, 2019). Recently, we described three EV-A71 isolates (S41, MS and C2 strains) that displayed differential neurotropic abilities in a unique symptomatic mouse model where mice develop progressive limb paralysis that correlates with presence of the virus in the CNS (Too et al., 2016). Interestingly, ability of these three isolates to invade the CNS in this mouse model correlated with their fitness in motor neuron NSC-34 cells. Comparison of their genome sequences allowed us to identify non-synonymous amino acid differences. Interestingly, none mapped into the main capsid protein VP1, which is involved in receptor-binding activity of the virus, suggesting that the differential fitness of these viruses is driven by viral determinants involved in post-receptor binding step(s) (Too et al., 2016). Here we studied the role of these non-synonymous mutations in EV-A71 fitness and virulence. We report the critical role of a single amino acid at position 149 in structural protein VP2 in the neurovirulence of EV-A71 in mice.

## Materials and Methods

### Ethics Statement

All the animal experiments were carried out under the guidelines of the National Advisory Committee for Laboratory Animal Research (NACLAR) in the AAALAC-accredited NUS animal facilities. The animal experiments were approved under the NUS Institutional Animal Care and Use Committee (IACUC) protocol numbers 070/10, 139/12 and R16-0136. Non-terminal procedures were performed under anesthesia, and all efforts were made to minimize suffering.

### Mammalian Cell Lines and Virus Strains

Human rhabdomyosarcoma (RD) cells (ATCC^®^ CCL-136™), NSC-34 mouse motor neuron-like hybrid cells (Cellutions Biosystems, CLU140), SK-N-SH (ATCC^®^ HTB-11™), SH-SY5Y human neuroblastoma cells (ATCC^®^ CRL-2266™), and C2C12 mouse myoblast cells (ATCC^®^ CRL-1772™), were employed in this study. Cells were cultured according to the supplier’s instructions.

The Enterovirus 71 strain used in this study is non-mouse-adapted EV-A71 Strain 41 (S41) (5865/SIN/00009, Accession No.: AF316321) (Singh et al., 2002). The infectious clone of S41 strain was described previously (Gunaseelan et al., 2019). All the virus stocks were made in RD cells and the viral titers were determined by plaque assay using RD cells.

### Enterovirus 71 Infection of Cell Lines

EV-A71 infection was performed at various multiplicity of infection (MOI) depending on the cell line and as indicated in the figure legends. Infection was carried out at 37°C and 5% CO_2_ for 1 h. The monolayers were washed with PBS and incubated with DMEM (Gibco) supplemented with 2% FBS (Gibco).

For transfection experiments, EV-A71 viral genome was extracted from infected cell culture supernatant using QIAamp Viral RNA Mini Kit (Qiagen), according to the manufacturer’s instructions. Viral RNA was diluted to 0.25 μg with OptiMEM (Invitrogen) in a total volume of 50 μL, and incubated for 5 min at room temperature (RT). After incubation, 1 μL of Lipofectamine 2000 (Invitrogen) was added to the RNA mixture and topped up to 100 μL, followed by 30 min incubation at RT. NSC-34 cell suspension (10^5^ cells per 400 μL) was mixed with 100 μL of transfection mixture and added into each well. The culture supernatant was harvested at different time-points for viral titer determination.

To generate virus from the infectious clones, viral RNA was obtained using the MEGAscript T7 Transcription Kit (Thermo Fisher Scientific). Viral RNA (2 μg) was transfected into RD cells using Lipofectamine 2000 transfection reagent (ThermoFisher) according to the manufacturer’s protocol. At 24 h post-transfection, the virus-containing supernatant was transferred to infect RD cells (in a T-75 flask), and passaged once 24 h later. Virus was then harvested at 24 h post-infection (h.p.i) and concentrated by polyethylene glycol (PEG) precipitation. Briefly, the supernatant was centrifuged at 500 × *g* for 20 min at 4°C. PEG 7% (w/v) in 2.3% (w/v) NaCl was then added, and the solution was stirred overnight at 4°C. The solution was centrifuged at 10,000 × *g* for 20 min at 4°C. The virus-containing pellet was resuspended in PBS (8 mL per T-175 flask used for propagation). The virus stock was stored at –80°C and the viral titer was determined by plaque assay.

### Virus Quantification by Plaque Assay

RD cells (10^5^ cells per well) were seeded onto 24-well plates. Culture supernatant from EV-A71-infected samples was serially diluted (10-fold) with 2% FBS-DMEM prior to infection. The cell monolayer was incubated with 100 μL of the diluted viral suspension for 1 h at 37°C and 5% CO_2_. The cells were then washed twice with PBS and replaced with 1 mL 2% FBS-DMEM and 1% carboxymethyl cellulose (CMC, Sigma Aldrich). After incubation for 3 days at 37°C and 5% CO_2_, the infected monolayers were fixed with 4% paraformaldehyde and stained with 0.1% crystal violet solution (Sigma Aldrich). The number of virus plaques were scored visually and viral titers were expressed as plaque-forming units (PFU) per milliliter (PFU/mL).

### Kinetic Profile of Virus Infection

RD (2 × 10^5^ cells per well), NSC-34 (3 × 10^5^ cells per well), SK-N-SH (2 × 10^5^ cells per well) and SH-SY5Y cells (2 × 10^5^ cells per well) were seeded onto 24-well plates and infected with 200 μL of virus diluted in 2% FBS-DMEM for 1 at 37°C at MOI indicated in the figure legends. After 1 h of incubation at 37°C and 5% CO_2_, the culture supernatant was removed and the cell monolayers were washed with PBS before 1 mL of culture medium was added. Supernatants were harvested at the indicated time points post-infection and quantified by plaque assay.

### Virus Quantification by Quantitative Real-Time Polymerase Chain Reaction

For quantification of intracellular viral RNA, NSC-34 cells (5 × 10^6^) were seeded onto T-25 flasks and infected with EV71 at the indicated MOI. At the indicated time-points, the supernatants were removed, and the cells were washed twice with PBS. Upon trypsinization for 1 min at 37°C and 5% CO_2_, cells were washed thrice with DMEM and gently pelleted by centrifugation at 500 × *g*.

For viral attachment assay, cells were infected at 4°C for 1 h, before washing twice with PBS. The cells were dislodged by gentle flushing with DMEM, and pelleted by centrifugation.

Viral RNA was extracted from cell pellets via the phenol-chloroform method. Briefly, 1 mL of TRIzol reagent (Life Technologies) was added to the cell pellet. Chloroform (200 μL) was added, and the samples were mixed thoroughly before being incubated at room temperature (RT) for 3 min. Samples were then centrifuged at 12,000 rpm for 15 min at 4°C to obtain the RNA-containing aqueous phase. Isopropanol was added to precipitate RNA from the aqueous phase and subsequently isolated using the RNeasy mini kit (Qiagen), following the manufacturer’s instructions. The RNA pellet was eluted from the column using nuclease-free water (Life Technologies). Purified RNA was treated with DNAse I (Sigma-Aldrich) to remove genomic DNA, and then quantitated using Nanodrop. Complementary DNA (cDNA) was synthesized from RNA using iScript cDNA synthesis kit (Bio-Rad). PCR was then carried out using the 7,500 real-time polymerase chain reaction (RT-PCR) system (Applied Biosystems). The iTaq Universal SYBR green supermix (Bio-Rad) was used in the relative real-time SyBr green RT-PCR reaction quantification assay of EV-A71 VP1 fragment, using mouse glyceraldehyde 3-phosphate dehydrogenase (GAPDH) as an internal control (Supplementary Table 1). Forty cycles of amplification were completed with each cycle consisting of 95°C for 15 s and 60°C for 1 min. EV71 RNA levels (*Ct*-values) were normalized to GAPDH RNA levels for each of the infected and uninfected samples (expressed as a ratio), before applying the comparative C_*T*_ method 2^ΔΔCT^ to calculate the fold change between infected and uninfected samples. Data were expressed as the mean ± SD of technical triplicates.

### Generation of Mutant Infectious Clones

The mutant infectious clones (Table 1) were generated using the Quikchange II XL site-directed mutagenesis kit (Agilent) using S41 infectious clone as template. Mutations were introduced in the primer sequences (Supplementary Table 1). The recombinant plasmids were then transformed into One Shot TOP 10 chemically competent *E. coli* (Thermo Fisher Scientific) according to the manufacturer’s protocol. Plasmids were extracted and purified using the QIAprep spin miniprep kit (Qiagen) and the presence of the desired mutation was verified by sequencing.

**TABLE 1:** Amino acid substitutions introduced in the EV-A71 S41 infectious clone.

Mutant	Protein	Position	Amino acid substitution	Nucleotide changes
VP2 I149K	VP2	149	Isoleucine to Lysine	ATA to AAG
3AV62M	3A	62	Valine to Methionine	GTG to ATG
3AT66A	3A	66	Threonine to Alanine	ACT to GCC
3CI79T	3C	79	Isoleucine to Threonine	ATT to ACC
3CM182E	3C	182	Methionine to Glutamic acid	ATG to GAG
3DC113H	3D	113	Cysteine to Histidine	TGC to CAC
3DA436T	3D	436	Alanine to Threonine	GCA to ACA

### Immunofluorescence Assay

NSC-34 cells (3 × 10^5^ cells) were seeded onto sterile coverslips and infected for 1 h with various EV-A71 strains at MOI 10. At the indicated time-points p.i, cells were fixed with ice-cold methanol for 20 min before incubation with AF647-conjugated mouse anti-dsRNA antibody (Engscicons, 1:400 dilution) or mouse anti-EV71 monoclonal antibody (Merck MAB979, 1:1,000 dilution). After washing with PBS, the cells were incubated with anti-mouse Alexa Fluor 488 (Millipore, 1 mg/mL, 1:500 dilution). NucBlue^®^ Live ReadyProbes^®^ Reagent (Life Technologies) was used to stain cell nuclei. The samples were mounted onto glass slides with 1, 4-diazabicyclo[2.2.2]octane (DABCO) (Life Technologies) before the edges of the coverslips were sealed with a thin film of nail varnish. The samples were viewed and representative images were captured using the microscope Olympus IX81.

### Murine SCARB2 siRNA Knockdown

Murine anti-SCARB2 siRNA and non-targeting controls (NTC) (Dharmacon) were prepared at 50 nM using DharmaFECT 1 Transfection Reagent (Dharmacon) at a final dilution of 1:1,000. After incubating for 30 min, the siRNAs were reverse transfected into NSC-34 cells (1.2 × 10^5^ cells) or C2C12 cells (9 × 10^4^ cells). Cells were incubated for 2 days to achieve siRNA-mediated knockdown.

### Western Blot

Cell lysates were prepared with the Mammalian Protein Extraction Reagent (M-PER; Pierce) with 1% Halt Protease Inhibitor Cocktail (Pierce) and 5 mM ethylenediaminetetraacetic acid (EDTA). Cell lysates were electrophoresed on 10% SDS-PAGE gel and transferred to nitrocellulose membranes (Bio-Rad Laboratories) using the Trans-Blot Turbo Transfer System (Bio-Rad Laboratories). Membranes were probed with primary antibodies against the viral VP0 protein (Merck Millipore, #MAB979), SCARB2 (Abcam, #ab176317) and Histone H2B (Thermo Fisher Scientific, # PA141058) overnight at 4°C. Goat anti-mouse 488 (Thermo Fisher Scientific, #A-11001) and goat anti-rabbit StarBright Blue 700 (Bio-Rad Laboratories, #12004162) served as secondary antibodies. Blot membranes were scanned using the ChemiDoc MP imaging system (Bio-Rad Laboratories). Band intensities were quantified using Image Lab Software (Bio-Rad Laboratories).

### Mouse Experiments

AG129 (deficient in Type I and II IFN receptor) mice (B&K Universal, United Kingdom) were bred and housed in pathogen-free conditions in individual ventilated cages. Infection of 2-week-old mice was performed via the intraperitoneal (ip) route with EV-A71 strains, each at a concentration of 10^7^ PFU per mouse in 200 μL of PBS. Uninfected control mice were administered with sterile PBS. Mice were weighed and observed daily for a period of 20 days. Clinical disease and symptoms were scored as follows: 0, healthy; 1, ruffled hair and hunchbacked appearance; 2, limb weakness; 3, paralysis in one limb; 4, paralysis in two limbs. Upon observation of two-limb paralysis, animals were promptly euthanized for ethical reasons.

For virus load determination, mice were euthanized at the indicated time-points and were systemically perfused with 50 mL of PBS. The front and hind limb muscles, spinal cords and brains were harvested, weighed and homogenized with a mechanical homogenizer (Omni, United States) in 1 mL DMEM. The homogenates were clarified by centrifugation at 14,000 rpm for 10 min at 4°C. Clarified supernatants were passed through a 0.22μm syringe-driven filter unit (Millipore) before serial dilution was carried out for plaque assay. Viral titers were expressed as PFU per gram of tissue.

For histology analysis, euthanized mice were systemically perfused with 50 mL of sterile PBS and 30 mL of 10% neutral buffered formalin (NBF) (Sigma-Aldrich). The hind limbs, front limbs, spinal cords and brains were harvested and incubated in NBF at RT for 72 h. Bony tissues were decalcified with 10% EDTA (1st Base) for 2 days and washed with deionized water for 1 h on a shaker. Fixed tissues were paraffin embedded, sectioned and stained with hematoxylin and eosin (H&E) before observation under a light microscope (Leica 3-way histology microscope).

### Statistics

All statistical analyses were carried out using Graphpad Prism software 5.02 (GraphPad Software, Inc.). Data were expressed as mean ± standard deviation (SD), or mean ± standard error of the mean (SEM). Kaplan-Meier survival curves and clinical score curves were analyzed using a log rank test and the Kruskal Wallis test with Dunn’s test as a *post-hoc* test, respectively. A two-way ANOVA with Bonferroni correction or a two-tailed unpaired *t*-test were used to analyze differences between groups. Statistical significance is denoted as *p < 0.05, ^**^p < 0.01, ^***^p < 0.001.

## Results

### VP2 I149K Mutant Does Not Productively Infect Motor Neuron NSC-34 Cells

We previously reported a number of non-synonymous amino acid changes between neurotropic S41 and myotropic MS and C2 strains (Too et al., 2016). These amino acid differences that change the nature of the amino acid are expected to affect the protein conformation and possibly its function. To evaluate to what extent these amino acid differences play a role in the fitness and virulence of EV-A71, site-directed mutagenesis was conducted to introduce each amino acid substitution into the S41 genome (Table 1).

**FIGURE 1:**
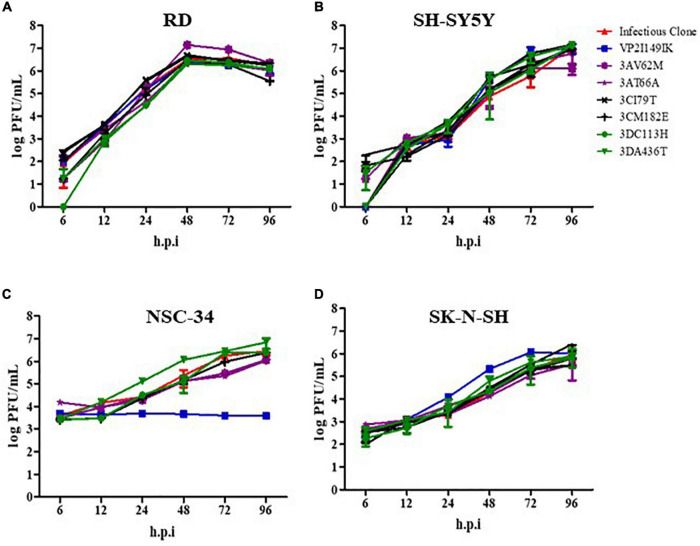
Infection profiles of EV-A71 WT and mutant strains. RD **(A)**, SH-SY5Y **(B)**, NSC-34 **(C)** and SK-N-SH **(D)** cells were infected with the WT S41 strain and the various mutants at MOI of 0.001 (RD and SH-SY5Y), 0.1 (SK-N-SH), and 1 (NSC-34). Supernatants were harvested at the indicated time-points p.i., and the virus titers were quantified by plaque assay. One set representative of two independent experiments is shown. Data were expressed as mean ± SD.

The infection profile of the mutants was determined in human rhabdomosarcoma RD, human neuroblastoma SK-N-SH and SH-SY5Y cell lines, and mouse motor neuron NSC-34 cells, and these profiles were compared to that of WT S41 strain. Mutants 3AV62M, 3AT66A, 3CI79T, 3CM182E, 3DC113H, and 3DA436T were all able to replicate as efficiently as WT virus in all the cell lines tested (Figure 1). The virus titers increased over time, with a peak at 48 h.p.i (in RD cells) or 96 h.p.i (in SH-SY5Y, SK-N-SH, and NSC-34 cells). In contrast, virus titers obtained with VP2 I149K-infected NSC-34 cells did not increase over time (Figure 1C), suggesting that VP2 I149K mutant was impaired either in its ability to enter NSC-34 cells or at a subsequent step of the infection cycle including replication, virus assembly and/or exit of the infectious virus particles. Such defect appeared to be cell type-specific since the infection profile of VP2 I149K mutant in the other cell lines (RD, SK-N-SH, and SH-SY5Y) was comparable to that of WT S41 strain (Figure 1).

### VP2 I149K Mutant Is Impaired at the Cell Entry Step

**FIGURE 2:**
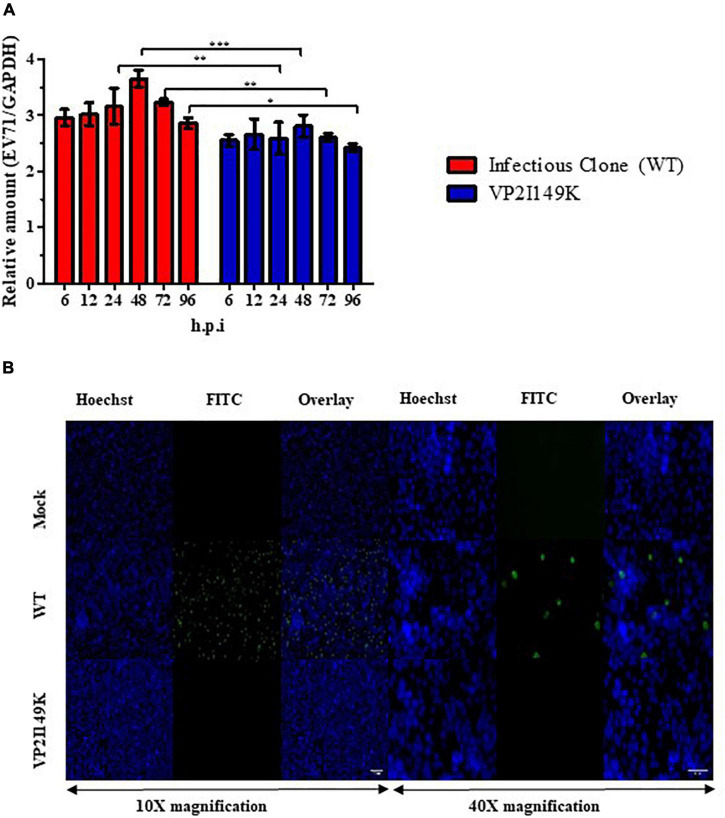
Replication of VP2 I149K mutant in NSC-34 cells. **(A)** Quantification of intracellular viral RNA. NSC-34 cells were infected with VP2 I149K mutant or WT S41 strain at MOI of 10. At the indicated time-points, infected cells were harvested and viral RNA was extracted and quantified by RT-qPCR. Intracellular viral RNA levels (*Ct*-values) were normalized to housekeeping GAPDH RNA levels. The fold change between infected vs. uninfected samples was calculated and data were expressed as the mean ± SD of technical triplicates. A two-way ANOVA with Bonferroni correction was used to evaluate statistical significance. *p < 0.05, **p < 0.01, *** *p* < 0.001. One representative set of two independent experiments is shown. **(B)** Detection of dsRNA. NSC-34 cells were infected with WT S41 or VP2 I149K mutant at MOI of 10, or left uninfected (mock control). At 72 h.p.i, the cells were fixed and processed for immunostaining using antibodies specific to dsRNA (FITC green). Cell nuclei were stained with Hoechst 33342. The images were captured at 10× or 40× magnification. Representative images are shown. Scale bar denotes 100 and 40 μm for 10× or 40× magnifications, respectively.

To investigate the impaired ability of VP2 I149K mutant to productively infect NSC-34 cells, we quantified the amounts of intracellular RNA over time, and compared to the WT strain. In NSC-34 cells infected with WT S41 strain, the intracellular viral VP1 RNA levels increased over time with a peak at 48 h.p.i (Figure 2A). However, no increase in the intracellular viral VP1 RNA titers was observed with the VP2 I149K mutant, suggesting minimal or no replication.

To further investigate whether VP2 I149K mutant is able to replicate its viral genome in NSC-34 cells, we employed immunofluorescence assay (IFA) to detect the presence of dsRNA, an intermediate RNA species generated during replication. Whereas RNA signal was readily detected in the cytoplasm of NSC-34 cells infected with WT S41 strain, no signal was seen with cells infected with VP2 I149K mutant, thus suggesting that the mutant is unable to replicate in NSC-34 cells (Figure 2B).

**FIGURE 3:**
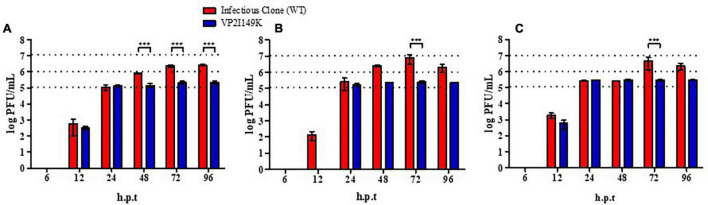
Infection profile of WT and VP2 I149K mutant upon reverse-transfection. **(A)** 25 ng, **(B)** 50 ng, or **(C)** 125 ng of viral RNA purified from WT or VP2 I149K mutant were reverse-transfected into NSC-34 cells. At the indicated time-points p.i., supernatants were harvested and the virus titers were determined by plaque assay in RD cells. One representative set of two independent experiments is shown. Data were expressed as mean ± SD. A two-way ANOVA with Bonferroni correction was used to evaluate statistical significance. ^***^p < 0.001.

Since VP2 is a structural protein that is proposed to be involved in virus uncoating (Dang et al., 2014), we hypothesized that the mutant may be impaired in this early step of the infection cycle, which may explain the inability to replicate its viral genome. To test this hypothesis, NSC-34 cells were reverse-transfected with a range of viral RNA quantities (25, 50, and 125 ng) prepared from the WT or VP2 I149K mutant. Viral titers in the culture supernatant were then monitored over time. This approach allows bypass of the entry step (including attachment at the cell surface, receptor-binding and uncoating), and evaluation of the ability of the mutant to replicate in NSC-34 cells compared to the WT. Whereas the WT viral titers kept increasing over time, the titers obtained upon transfection with the RNA prepared from VP2 I149K mutant reached a plateau at 24 h post-transfection onward (Figure 3). This observation suggested that upon transfection, the VP2 I149K virus was able to replicate productively in the transfected NSC-34 cells. However, the virus progeny released from the cells was unable to infect a second round of NSC-34 cells, strongly supporting that the VP2 I149K mutant is impaired at the entry step. To further confirm this hypothesis, the intracellular viral RNA levels were monitored over a 24-h period post-transfection, which represents the first infection cycle. Comparable profiles were observed with both the WT and VP2 I149K mutant, whereby the viral VP1 RNA levels increased from 6 to 12 h, followed by a decrease (Supplementary Figure 1). This result thus confirmed that upon transfection of the viral RNA, VP2 I149K mutant is able to replicate its viral genome inside NSC-34 cells, and generate virus progeny as efficiently as the WT strain.

**FIGURE 4:**
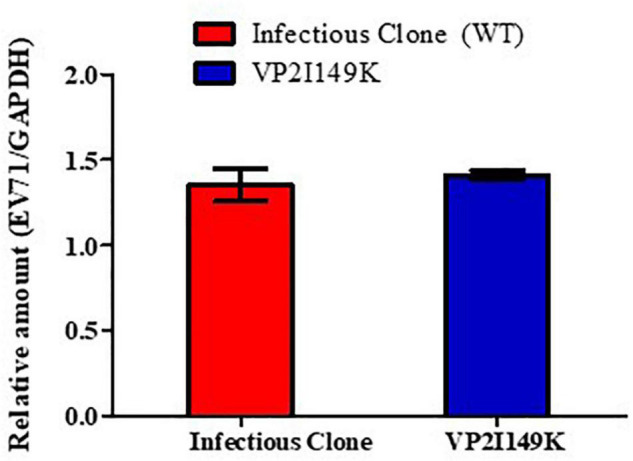
Binding assay. NSC-34 cells were infected with either WT or VP2 I149K mutant (MOI of 10) at 4°C for 1 h. The cells were washed and pelleted, and viral RNA was extracted and processed for RT-PCR. The viral RNA titers were normalized to GAPDH RNA levels. One representative set of two independent experiments is shown. Data were expressed as mean ± SD.

To further characterize the defect of VP2 I149K mutant at the entry step, we evaluated its ability to attach at the cell surface compared to WT S41. The WT and VP2 I149K mutant were allowed to interact with the surface of NSC-34 cells for 1 h at 4°C. The low temperature allows for virus attachment at the cell surface to occur, but prevents internalization. Following several rounds of washing, viral VP1 RNA levels were then quantified by RT-PCR. Similar levels of viral VP1 RNA levels were detected with the WT and VP2 I149K mutant (Figure 4). This result thus indicated that VP2 I149K amino acid substitution does not impair the ability of the virus to adhere to the cell surface, therefore suggesting that this viral determinant affects a downstream step of the entry process.

### Enterovirus 71 Strain 41 Virus Relies on Murine SCARB2 to Enter NSC-34 Cells

**FIGURE 5:**
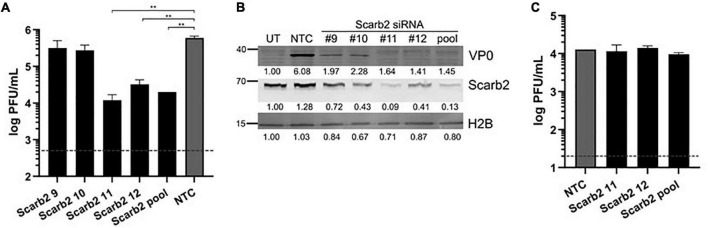
Effect of mSCARB2-siRNA knockdown on S41 WT infection in NSC-34 cells. NSC-34 cells were reverse-transfected with mSCARB2 siRNA (pool or deconvoluted) or siRNA non-treated control (NTC), followed by infection with WT S41 virus at MOI of 10 **(A,B)** or transfection with purified S41 viral genome **(C)**. **(A,C)** Viral titers in the culture supernatants sampled at 48 h **(A)** and 18 h **(C)** post-infection were determined by plaque assay. The horizontal dashline indicates the detection limits of 500 PFU/mL **(A)** and 20 PFU/mL **(C)**, respectively. Two-tailed student *t*-test was conducted with **p < 0.01. Data were expressed as mean ± SD. **(B)** SCARB2 and viral VP0 in cell lysates from experiment **(A)** were probed by immunoblotting. Histone H2B was used as loading control. Band intensities were normalized to untreated non-infected control (UT).

Since VP2 I149K mutant was specifically impaired in the ability to enter murine NSC-34 cells but not human muscle and neuroblastoma cell lines, we hypothesized that the I149K substitution specifically impairs the ability of the virus to engage with its murine receptor for successful entry. EV-A71 is known to enter human cells via SCARB2 (hSCARB2), whereas affinity of EV-A71 for the murine SCARB2 counterpart (mSCARB2) was found to be weaker (Dang et al., 2014; Kobayashi and Koike, 2020). A previous study reported that amino acids 98E, 145A, and 169F in VP1 improved EV-A71 affinity for mSCARB2, thereby allowing greater infectivity of mouse cell lines (Victorio et al., 2016). Strain S41 harbors an aspartic acid residue at position 98 (98E) in its VP1 protein. Thus, we examined whether S41 virus exploits mSCARB2 to enter NSC-34 cells. First, siRNA knockdown of mSCARB2-encoding gene in NSC-34 cells led to significant reduction in viral titers (Figure 5A), which correlated with lower intracellular production of viral proteins (Figure 5B), indicating that mSCARB2 is a pro-viral host factor in NSC-34 cells. Similar observations were made with the murine muscle cell line C2C12 that is permissive to S41 infection (Supplementary Figure 2), indicating that the use of mSCARB2 by this virus strain is not limited to NSC-34 cells. To ascertain the role of mSCARB2 during the entry step of the virus life cycle in NSC-34 cells, mSCARB2 siRNA-knocked down NSC-34 cells were transfected with S41 viral genome. Culture supernatants were sampled after a single round of replication and showed no difference in viral titers compared to the NTC control (Figure 5C). These results thus strongly supported that S41 virus exploits mSCARB2 to enter murine cells, thereby implying that the I149K substitution in VP2 specifically impairs mSCARB2-mediated viral entry.

### The VP2 I149K Mutant Is Unable to Invade the Central Nervous System in Mice and Cause Symptomatic Infection

**FIGURE 6:**
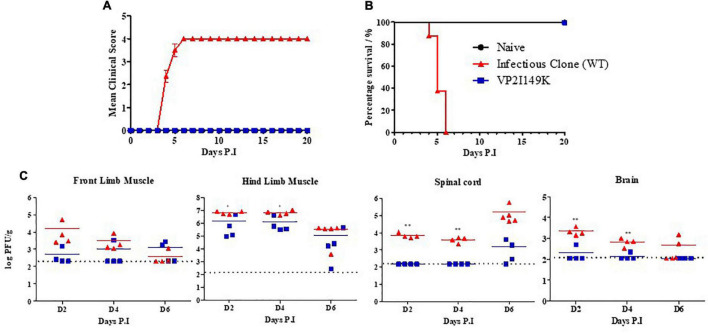
Infection of WT or VP2 I149K mutant in a symptomatic mouse model. Two-week-old AG129 mice (*n* = 8–10) were infected intraperitoneally with 10^7^ PFU of WT or VP2 I149K mutant. Uninfected control mice were given PBS instead (*n* = 3). Mice were monitored over a 20-day period. **(A)** Clinical scores (*n* = 8–10) were defined as follows: 0, healthy; 1, ruffled hair and hunchbacked appearance; 2, limb weakness; 3, paralysis in one limb; 4, paralysis in two limbs. Mice were euthanized when they reached a score of 4. **(B)** Survival rate of mice. One representative set of two independent experiments is shown. Data were expressed as mean ± SEM. **(C)** Virus titers in front and hind limb muscles, spinal cords and brains (*n* = 3–5 per time point) were determined at the indicated time-points by plaque assay. Individual values are shown. Means are represented by solid lines. The dotted line represents the limit of detection of the assay. Values below the limit of detection were assigned an arbitrary value equivalent to the limit of detection. One representative set of three independent experiments is shown. A two-tailed student’s *t*-test was carried out to evaluate statistical significance. *p < 0.05, ^**^p < 0.01.

We next tested whether the inability to infect motor neuron NSC-34 cells would translate into the inability to cause symptomatic infection in mice. Two-week-old AG129 mice were infected with WT S41 or VP2 I149K mutant. Similar to our previous report (Too et al., 2016), mice infected with WT S41 started to exhibit clinical symptoms at day 4 p.i, and developed progressive limb paralysis (one-limb to two-limb paralysis) (Figures 6A,B). By day 6 p.i, all the animals had displayed two-limb paralysis and were euthanized to minimize suffering. In contrast, mice infected with VP2 I149K mutant did not exhibit any clinical symptoms or signs of disease throughout the course of the experiment (Figures 6A,B).

Furthermore, the viral titers in the limb muscles were comparable between both infected groups, although the viral titers in the hind limb muscles were statistically significantly different at days 2 and 4 p.i (*p* < 0.05) (Figure 6C). In sharp contrast, the viral titers in the spinal cords were much higher in mice infected with WT S41 compared to VP2 I149K-infected mice (Figure 6C). The titers obtained with VP2 I149K-infected mice were indeed below the limit of detection at days 2 and 4 p.i. The viral titers at day 6 p.i. in the spinal cords from the WT-infected mice were higher than those measured in VP2 I149K-infected mice, although they did not reach statistical significance likely due to the limited number of mice per group. Similarly, viral titers in the brains of mice infected with the WT strain were statistically higher at days 2 and 4 p.i compared to those measured in the brain from VP2 I149K-infected mice, with viral titers of all the mice below the limit of detection, except for one mouse at day 2 and one mouse at day 4 p.i (Figure 6C).

**FIGURE 7:**
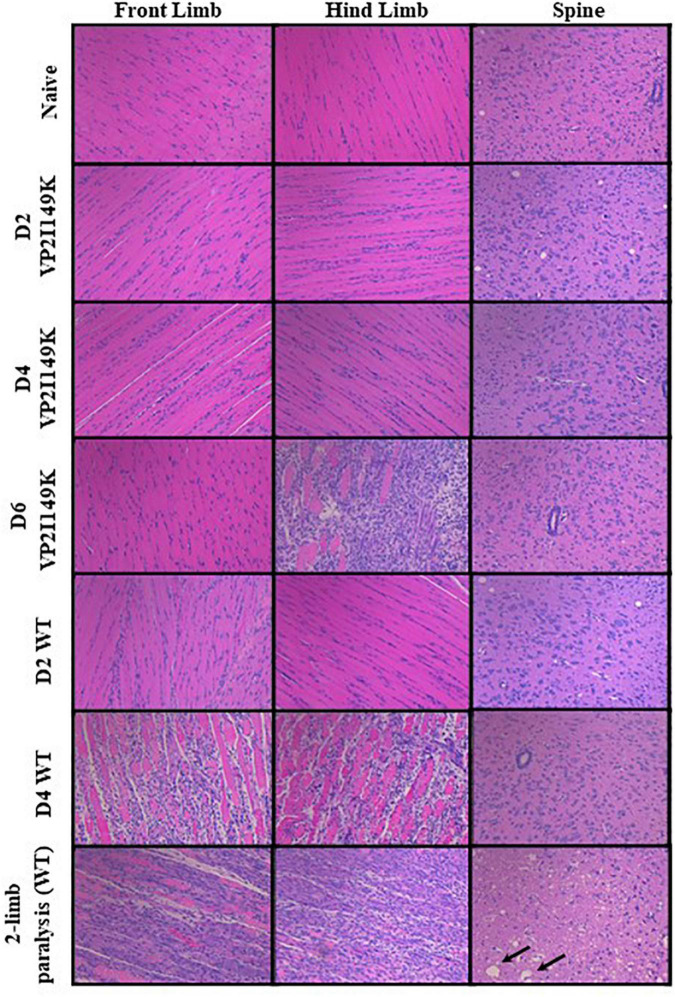
Histopathological analysis of virus-infected mice. Two-week-old AG129 mice (*n* = 3) were infected intraperitoneally with WT or VP2 I149K mutant. Uninfected control mice were administered with PBS instead (naïve). Mice were euthanized upon observation of two-limb paralysis, or at the indicated time-points, and organs were harvested and processed for H&E staining. Black arrows indicate neuropil vacuolation and neuronal degeneration in the anterior horn region of the spinal cord. All observations were made at 20× magnification. The scale bar denotes 100 μm. Representative images are shown.

The extent of histopathological damage observed in mice infected with WT or VP2 I149K mutant was analyzed at days 2, 4 or 6 p.i or once two-limb paralysis was observed. VP2 I149K infection did not cause any myositis except at day 6 p.i in the hind limbs (Figure 7). This was in contrast to WT-infected mice where severe myositis with neutrophilic infiltration was observed in both the front and hind limbs from day 4 p.i and in mice with two-limb paralysis. Furthermore, VP2 I149K-infected mice did not exhibit any neuropil vacuolation or neuronal loss as observed in the spinal cords from WT-infected mice. Thus, histopathological injury caused by VP2 I149K mutant was clearly less severe than observed in WT-infected mice.

Taken together, the data indicate that the VP2 I149K mutant is as capable as the WT strain of replicating in murine skeletal muscles of mice but is impaired in its ability to invade the CNS and cause symptomatic disease in the AG129 mouse model. These *in vivo* findings thus correlated well with the *in vitro* observations.

## Discussion

In this study, we showed that the single amino acid substitution I149K in VP2 was sufficient to abrogate the ability of EV-A71 S41 strain to productively infect murine motor neuron-like NSC-34 cells. We provided several lines of experimental evidence supporting that this defect was attributed to the inability of the mutant to enter these cells, downstream of the cell attachment step. Importantly, these *in vitro* observations correlated with the impaired ability of VP2 I149K mutant to invade the CNS in mice, resulting in asymptomatic disease. These findings therefore support a critical role of the amino acid 149 in VP2 in conferring neurovirulence *in vivo*.

Viral entry comprises several steps, including virus attachment, internalization, uncoating and RNA genome release (Ku et al., 2015). EV-A71 is known to use human SCARB2 and PSGL-1 receptors for cell entry, with the main capsid protein VP1 involved in host receptor binding (Kobayashi and Koike, 2020). We have shown here that S41 virus likely uses mSCARB2 to enter NSC-34 cells. Given that VP2 I149K mutant virus could infect human skeletal muscle and neuroblastoma cell lines as effectively as WT S41 strain, our observations suggested that the VP2 I149K substitution specifically impairs mSCARB2-mediated viral entry. The binding assay result revealed that both the WT and VP2 I149K mutant could bind at the cell surface equally well, thereby excluding an overt defect in attachment at the cell surface and receptor binding for the mutant. Homology remodeling and structural studies have placed residue 149 of VP2 on the surface-exposed region of the EF loop (residues 136–150), and indicated that the EF loop interacts with VP1 during hSCARB2-mediated entry to facilitate viral uncoating (Zaini et al., 2012; Xu et al., 2014; Zhou et al., 2019). Our data here suggests that while the VP2 I149K mutation does not affect hSCARB2-mediated entry and uncoating (in human cell lines), it critically impacts mSCARB2-mediated entry/uncoating into NSC-34 cells. Interestingly, while substituting Isoleucine (I) with Lysine (K) at position 149 in VP2 drastically impaired the ability of S41 to enter NSC-34 cells, the presence of a Lysine residue at this position in VP2 of other EV-A71 strains (MS and C2 strains) did not prevent these from productively infecting NSC-34 cells (Too et al., 2016). This observation thus suggests that other residues in VP2 and/or VP1 interact with 149K in VP2 to allow these virus strains to successfully complete the viral entry step into NSC-34 cells.

A number of studies have indicated that the nature of amino acid 149 in VP2 significantly impacts EV-A71 fitness *in vitro* and/or *in vivo*. Mutation at this position was indeed reported to occur during mouse-adaptation of EV-A71 in the brain, suggesting that this amino acid substitution can confer neurovirulence in mice (Wang et al., 2004). The amino acid at position 149 in VP2 also plays a role in the virus entry via human PSGL-1 receptor expressed on previously non-susceptible mouse cells (Miyamura et al., 2011). Another study showed that the VP2 K149M mutation enhances viral release, resulting in increased viral titers in murine Neuro-2a cells (Huang et al., 2012). *In vivo* however, the VP2K149M mutation was insufficient in enhancing virulence and mortality in mice. However, introduction of the VP1 Q145E mutation together with the VP2 K149M mutant led to augmented binding to Neuro-2a cells and increased mortality in mice (Huang et al., 2012). In yet three other studies, the VP2 K149M/I mutations resulted in increased infectivity in Chinese hamster ovary (CHO) cells, but did not increase virulence in mice (Chua et al., 2008; Zaini et al., 2012; Zaini and McMinn, 2012). More recently, the VP2 K149I mutation was reported to increase virulence of the EV-A71 C4 strain in mice (Xu et al., 2017), which is directly congruent with our own findings. Indeed, we found that the reverse amino acid substitution, VP2 I149K, in a neurovirulent EV-A71 strain led to a complete loss of virulence *in vivo*, whereby 2-week-old AG129 mice infected with this VP2 I149K mutant remained healthy and exhibited no limb paralysis. Importantly, and uniquely, while comparable viral titers were measured in the limb muscles, much lower titers were found in the spinal cords and brains of the mice infected with this mutant virus. This result supported that the mutant was specifically impaired in its ability to invade the CNS of these mice, but was not affected in its ability to replicate in muscle cells, thus pointing to a specific role for VP2 I149K in EV-A71 neurovirulence. In this AG129 mouse model, these observations further support that EV-A71 invades the CNS by infecting motor neurons at the neuromuscular junctions.

Together, our study provides additional evidence of a critical role of amino acid 149 in VP2 in EV-A71 neurovirulence. It also offers further insights into the involvement of this residue located in the EF loop of VP2, in SCARB2-mediated viral entry step. Interestingly, a recent study has reported the role of another amino acid (Ile 135) located in VP2 EF loop in PSGL1-mediated entry and in viral neuroinvasion in a SCID mouse model of EV-A71 infection (Sun et al., 2019), further supporting the role of the VP2 EF loop in the viral entry step in neuronal cells. Targeting the entry step represents an attractive approach for the development of antivirals and vaccines. While much effort has focused on the main capsid VP1, our study further supports previous vaccine studies indicating that the VP2 EF loop (particularly the epitope spanning residues 141–155), is immunodominant and protective (Xu et al., 2014, 2015).

## Data Availability Statement

The original contributions presented in the study are included in the article/Supplementary Material, further inquiries can be directed to the corresponding author/s.

## Ethics Statement

The animal study was reviewed and approved by the NUS Institutional Animal Care and Use Committee (IACUC).

## Author Contributions

HY, CWHC, EWC, ZL, QN, and BY performed the experiments. JC and VC provided materials. YH and SA designed the experiments and wrote the manuscript. All authors contributed to the article and approved the submitted version.

## Conflict of Interest

The authors declare that the research was conducted in the absence of any commercial or financial relationships that could be construed as a potential conflict of interest.

## Publisher’s Note

All claims expressed in this article are solely those of the authors and do not necessarily represent those of their affiliated organizations, or those of the publisher, the editors and the reviewers. Any product that may be evaluated in this article, or claim that may be made by its manufacturer, is not guaranteed or endorsed by the publisher.
