# Supplementary material for: A Single Amino Acid Substitution in Structural Protein VP2 Abrogates the Neurotropism of Enterovirus A-71 in Mice
Source: Front Microbiol. 2022 Mar 17;13:821976. doi: 10.3389/fmicb.2022.821976 (PMC8969769; doi:10.3389/fmicb.2022.821976)
Supplement: Supplementary file 1 [file Table_1.DOCX]

**SUPPLEMENTAL INFORMATION**

**Supplemental Table S1. Primer sequences used in this study.**

| **Target** | **Forward Primer** | **Reverse Primer** |
| --- | --- | --- |
|  | **Primers for** **qPCR (S41 strain)** |  |
| VP1 | GCACAGGTCTCAGTTCCGTT | CACGCCTGACATGCTTCAT |
| GAPDH | CATCATCTCCGCCCCTTCTG | TGGTCATGAGCCCTTCCACA |
|  | **Primers to generate the mutants** |  |
| VP2I149K | GCGCCAGGTTGTGTTTGCTTGTAAGGAGGGTGGCTG (36 nt) | CAGCCACCCTCCTTACAAGCAAACACAACCTGGCGC (36 nt) |
| 3AV62M | CAGTAGTGATGGATTGCATGACTAGCACTGCTCTATT (37 nt) | AATAGAGCAGTGCTAGTCATGCAATCCATCACTACTG (37 nt) |
| 3AT66A | CGACTGCCACCACAGTGGCGATGGATTGCACGACT (35 nt) | AGTCGTGCAATCCATCGCCACTGTGGTGGCAGTCG (35 nt) |
| 3CI79T | GGTGATATCTCTAAATTTTTCATTGGTATCTAGTGTCACCAATGTGAGTTCC (52 nt) | GGAACTCACATTGGTGACACTAGATACCAATGAAAAATTTAGAGATATCACC (52 nt) |
| 3CM182E | CCATTGGATCTCACCTTGCTCACTCGCAAAGTAGCTCCT (39 nt) | AGGAGCTACTTTGCGAGTGAGCAAGGTGAGATCCAATGG (39 nt) |
| 3DC113H | GGATACCCAGCACTAGTGTGGAGGTCTATTGCTTCCAG (38 nt) | CTGGAAGCAATAGACCTCCACACTAGTGCTGGGTATCC (38 nt) |
| 3DA436T | TCCAACTGGAACTGATCTAATTGTACTCACAACTTTTCATATTCAT (47 nt) | ATGAATATGAAAAGTTTGTGAGTACAATTAGATCAGTTCCAGTTGGA (47 nt) |

**Supplemental Figure S1**

**Figure S1. Intracellular viral RNA from WT S41 and VP2 I149K mutant over 24-hours post-transfection.** NSC-34 cells were transfected with viral RNA from WT S41 or VP2 I149K mutant. At the indicated time-points, intracellular viral RNA was extracted and quantified by RT-qPCR. Intracellular viral VP1 RNA titers were normalized to GAPDH RNA levels. One representative set of two independent experiments is shown. Data were expressed as mean ± SD.

**Supplemental Figure S2**


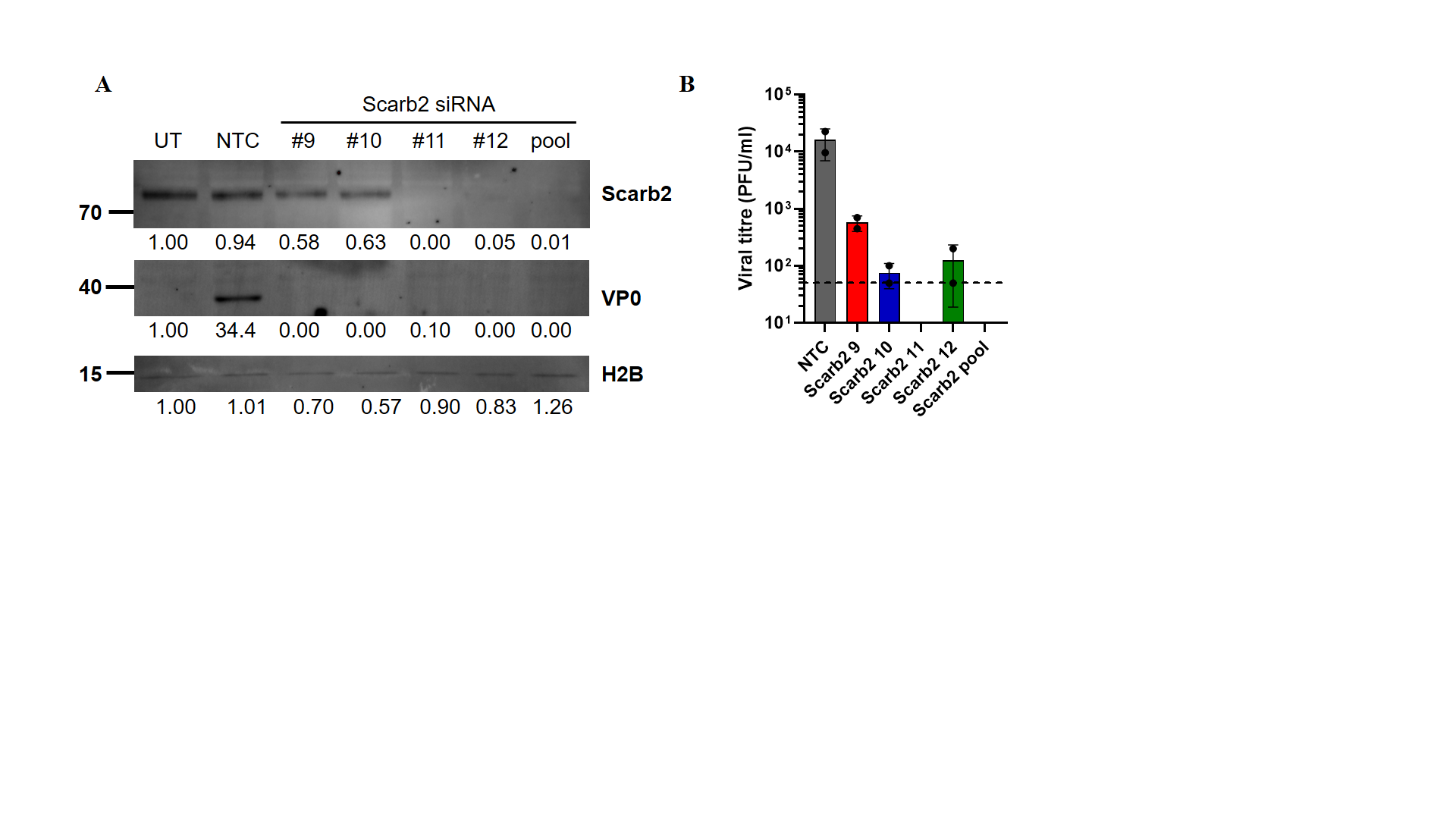


**Figure S2. siRNA-mediated knockdown of mSCARB2 in C2C12 cells.** Undifferentiated C2C12 were reverse-transfected with mSCARB2 siRNA (pool or deconvoluted) or non-template control (siNTC) siRNA, followed by infection with WT S41 virus at MOI 1. (A) SCARB2 and viral VP0 proteins in cell lysates were probed by immunoblot. Histone H2B was used as loading control. Band intensities were normalized to the untreated non-infected control. NTC: siNTC-treated control. (B) Viral titres in the culture supernatants sampled at 48 hours post-infection were determined by plaque assay. The horizontal dashline indicates the detection limit of 50 PFU/ml.
